# Figure Interpretation Assessment Tool-Health (FIAT-health) 2.0: from a scoring instrument to a critical appraisal tool

**DOI:** 10.1186/s12874-019-0797-6

**Published:** 2019-07-23

**Authors:** Reinie G. Gerrits, Niek S. Klazinga, Michael J. van den Berg, Dionne S. Kringos

**Affiliations:** Department of Public Health, Amsterdam UMC, University of Amsterdam, Amsterdam Public Health Research Institute, Meibergdreef 9, 1105 AZ Amsterdam, The Netherlands

**Keywords:** Knowledge translation, Policy makers, Science communication, Reporting tool, Reporting checklist, Dissemination, Stakeholder involvement

## Abstract

**Background:**

Statistics are frequently used in health advocacy to attract attention, but are often misinterpreted. The Figure Interpretation Assessment Tool–Health (FIAT-Health) 1.0 was developed to support systematic assessment of the interpretation of figures on health and health care. This study aimed to test and evaluate the FIAT-Health 1.0 amongst its intended user groups, and further refine the tool based on our results.

**Methods:**

Potential users (*N* = 32) were asked to assess one publicly reported figure using the FIAT-Health 1.0, and to justify their assessments and share their experience in using the FIAT-Health. In total four figures were assessed. For each figure, an expert on the specific topic (*N* = 4) provided a comparative assessment. The consistency of the answers was calculated, and answers to the evaluation questions were qualitatively analysed. A qualitative comparative analysis of the justifications for assessment by the experts and potential users was made. Based on the results, a new version of the FIAT-Health was developed and tested by employees (*N* = 27) of the National Institute for Public Health and the Environment (RIVM), and approved by the project’s advisory group. In total sixty-three participants contributed.

**Results:**

Potential users using the FIAT-Health 1.0 and experts gave similar justifications for their assessments. The justifications provided by experts aligned with the items of the FIAT-Health. Seventeen out of twenty-six dichotomous questions were consistently answered by the potential users. Numerical assessment questions showed inconsistencies in how potential users responded. In the evaluation, potential users most frequently mentioned that thanks to its structured approach, the FIAT-Health contributed to their awareness of the main characteristics of the figure (*n* = 14), but they did find the tool complex (*n* = 11). The FIAT-Health 1.0 was revised from a scoring instrument into a critical appraisal tool: the FIAT-Health 2.0, which was tested and approved by employees of the RIVM and the advisory group.

**Conclusion:**

The tool was refined according to the results of the test and evaluation, transforming the FIAT-Health from a quantitative scoring instrument into an online qualitative appraisal tool that has the potential to aid the better interpretation and public reporting of statistics on health and healthcare.

**Electronic supplementary material:**

The online version of this article (10.1186/s12874-019-0797-6) contains supplementary material, which is available to authorized users.

## Background

Statistics on health and healthcare gain much attention in public media. Figures are being published, cited, and summarized in press releases, newsletters, and news items every day [1, 2]. Moreover, in science communication, statistics are a persuasive tool for health policy advocacy [3–5]. Politicians, policy makers and journalists like to use so-called “killer stats”; headline-grabbing statistics that immediately grasp the attention of a specific audience. The complex character and methodological background, necessary to really understand these figures, often gets lost in translation [6–8]. Without the proper reporting of the background and methodology, figures are likely to be misinterpreted [9, 10]. Misinterpretation of these figures is problematic, as they may impact policy and practice [11, 12]. Spiegelhalter (2017) described the traditional information flows from statistical sources to the public [13]. First, statistics developed by (A) academic and industry scientific research are reported in scientific publications, or (B), commissioned analytic and survey research statistics are reported by policy makers, official statistic bureaus, NGO’s or other institutions. Second, press offices and communication departments report statistics to traditional media and online sources. Finally, through these sources the information is received by the public. In this communication flow, many questionable interpretation- and communication practices can occur, such as not reporting uncertainties, providing contexts or comparative perspectives, and providing relative but not absolute risk.

In the scientific community, many checklists and methods are available for the detailed appraisal and reporting of empirical studies, such as the EQUATOR guidelines [14]. Furthermore, recently the GATHER statement [15] was published to support the reporting of findings of Global Health Estimates targeted at researchers and decision makers. However, there is a lack of systematic methods for the reporting and appraisal of publicly reported statistics [16] i.e. statistics that were reported with the aim to inform the public or person who may apply the statistic in practice. Policy makers and civil society have other information needs than researchers when they interpret a figure [17, 18]. While researchers often need in-depth information on the underlying statistical methods, those with less technical knowledge have few methods for the interpretation of a published figure [19].

Therefore, we developed a method for the systematic appraisal of figures on health and healthcare: The Figure Interpretation Assessment Tool – Health (FIAT-Health) [20]. The FIAT-Health provides a systematic method for quantitatively assessing publicly reported figures on health and healthcare to be used by policy makers, managers, researchers, and the general public. The added value of this instrument is that its use requires little technical or methodological expertise. The first version, i.e. the FIAT-Health 1.0, consisted of 15 questions, which allow its user to better understand and interpret figures. In total 35 sub questions were included in the FIAT-Health covering factual dichotomous questions, to be answered by yes or no, assessment questions where the user assesses a characteristic of the figure on a scale from 1 to 5, and two final questions in which the user gives an overall assessment of the correctness of the figure and the appropriateness of the reporting of a figure on a scale from 1 to 4. Furthermore, a detailed explanation is provided for each question. The FIAT-Health was developed through consultation of 68 experts in four phases, and with the involvement of a sounding board (advisory group). The development of the FIAT-Health 1.0 was published elsewhere [20]. Face and content validity of the tool were established during the development of the FIAT-Health [20] but its usability has not been tested amongst its intended user groups, which is fundamental to the uptake of the tool in practice [21]. To further improve the usability of the FIAT-Health, the current study intends to test and evaluate the FIAT-Health 1.0 amongst its intended user groups, and further refine the tool based on our results. To find out to what extent users were able to make adequate assessments, we compared their assessments of figures with the FIAT-Health to an assessment made by experts on the specific topic who did not use the FIAT-Health.

## Methods

### Design

We used a qualitative content analysis approach in this study. Potential users were asked to test and evaluate the tool. To compare the justification of the assessments made with the tool, experts provided a comparative assessment. Based on the results, the FIAT-Health was refined and tested by employees of the National Institute for Public Health and the Environment (RIVM). A project advisory group was involved throughout the process to guide the refinement of the tool.

### Setting

The study took place in the Netherlands during February – August 2017, involving potential users from healthcare institutes from different regions.

### Figures used for testing

**Fig. 1:**
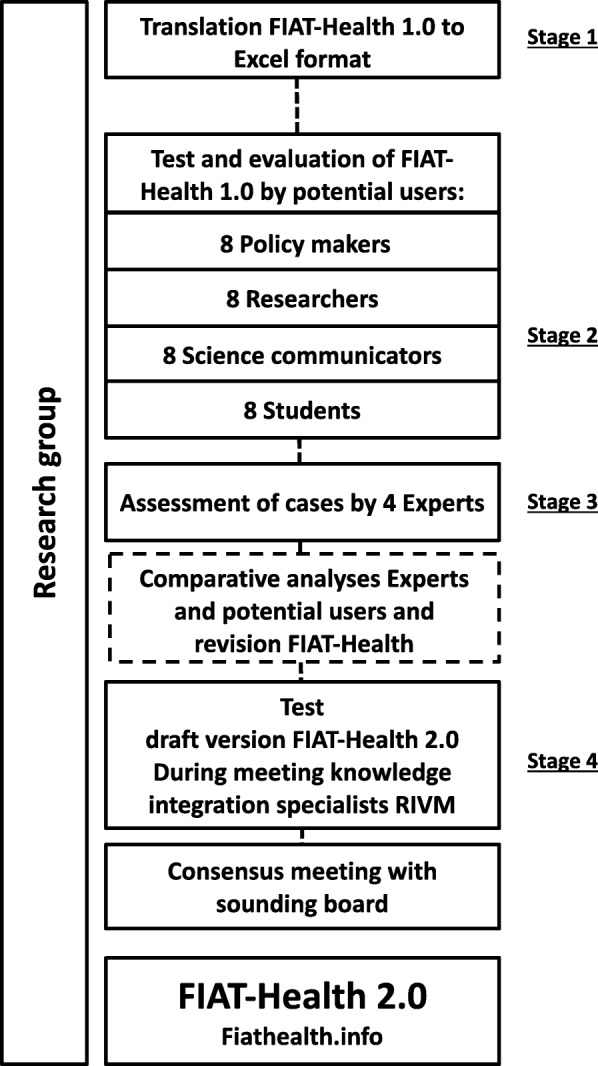
Data collection process

**Fig. 2:**
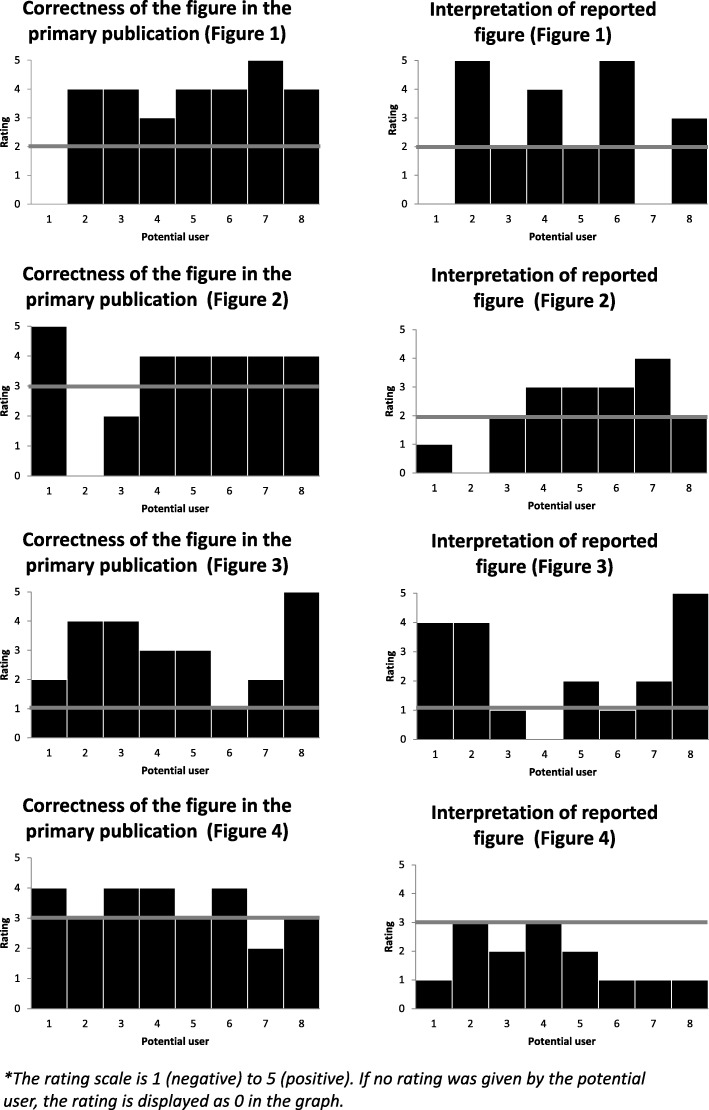
Assessments of the final assessment questions 14 and 15 per participant per figure, expert rating represented by the grey bars

Four different publicly reported figures were selected, including: the prevalence of Dutch people experiencing burnout complaints (figure 1) [22] the number of hours of intensive sports that reduces mortality risk (figure 2) [23] the financial profit from a decreasing number of Dutch smokers (figure 3) [24] and the number of premature deaths in people with dementia due to wrong medication (figure 4) [25].

The figures were selected based on a variation in primary publication, i.e. reports and peer-reviewed publications, the type of public report, and the expected quality of the publication as determined by the research group. Publications of which Amsterdam UMC, location Academic Medical Centre (AMC) and the National Institute for Public Health and Environment (RIVM) were primary authors, were not included given the affiliation of the authors. Publicly reported figures may be assessed in a primary publication. However, the figures used for testing the FIAT-Health 1.0 were all assessed in a secondary publication to include questions on the comparison between the reported figure and the primary publication.

Each potential user assessed one publicly reported figure. Each figure was assessed by two participants of each user group.

### Participants and recruitment

In the second stage, potential users were asked to test the FIAT-Health 1.0.

Four potential user groups were included in the study through purposeful selection: policy makers, researchers, communication officers, and students. Potential users were selected from the professional network of the project team, who had no previous knowledge of the study.

Potential users who accepted the invitation received an e-mail explaining the process of participation, and they received the FIAT-Health 1.0 in Excel format including the evaluation form that potential users were asked to fill in. The paper format of the FIAT-Health 1.0 was translated to an Excel format for the purpose of this study. The FIAT-Health 1.0 was put into an Excel format to allow for the structured use of the tool and to provide potential users with a systematic overview of their answers in the intended format. The FIAT-Health 1.0 in Excel format is included in Additional file 1.

Furthermore, potential users received the publicly reported figure (a newspaper or web publication), and the primary publication (a research report or peer-reviewed scientific publication). The potential users e-mailed their assessment and evaluation in the Excel file to RG, who collected all answers.

### Data collection process

Within the Excel file an evaluation form with six open-ended questions was included: *1. How do you experience the use of the FIAT-Health 1.0? 2. Which considerations had the largest impact on your evaluation regarding the correctness of the figure? 3. Which considerations had the largest impact on your assessment of reporting of the figure? 4. Did you experience any problems when using the FIAT-Health 1.0? 5. Were any important considerations missing in the FIAT-Health 1.0? 6. Do you have any suggestions for the improvement of the FIAT-Health 1.0?*

### Expert assessment

In the third stage, to compare the assessments by the potential users with the assessments by experts, four leading researchers from different universities, with a professorship in organisational psychology, sports medicine, health economics, and population health sciences respectively, were approached and asked to provide an expert assessment of one of the four figures that matched their expertise. The experts did not receive the FIAT-Health 1.0. They were asked to provide their assessment of the correctness of the figure and were asked to rate the figure with 1 to 5 stars (the last two assessment questions of the FIAT-Health) and justify their assessment. To date, no systematic method has been used for advising policy makers on figures, who mostly ask advice from leading researchers. As an expert assessment of a figure is current practice, we considered their assessment as the “gold standard” [26] for comparison with the assessment resulting from the FIAT-Health 1.0. Furthermore, their explanations for their assessments were used to compare with the justifications by the potential users.

Both potential users and experts participated voluntarily and were provided no individual incentives.

### Analyses

A qualitative comparative analysis of the justifications for assessment by the experts and the potential users was made. We applied a conventional content analysis method as described by Hsieh and Shannon (2005) [27]. All evaluations and assessments were read to gain an impression of the data. Second, from the explanation experts provided, justifications for their assessment were extracted. Third, justifications from all experts were compared and listed. Fourth, the potential users’ answers to evaluation questions 2 and 3 were coded into distinct justifications for assessment. Fifth, these justifications were categorised and compared to the expert justifications. Answers by experts and potential users to the final assessment questions on the correctness of the figure and the reporting of the figure were compared. If the justification used by the expert was identical to the justifications given by the potential users, justifications were considered to be comparative.

The evaluation by the potential users was derived from the answers to evaluation questions 1, 4, 5 and 6, and coded into common topics. All analyses were completed in Excel.

Moreover, to be able to see what questions may need revision, the agreement between participant answers on the numerical questions was calculated. Answers to dichotomous questions were considered inconsistent if the answer of two or more potential users deviated from the majority for at least two figures. The answers given to the assessment questions were considered as inconsistent if three or more answers deviated from the majority for at least two figures. One coder (RG) performed the analyses.

### FIAT-health 2.0

Finally, in the fourth stage of the study, we adapted the FIAT-Health and tested the FIAT-Health version 2.0. A first revision was presented to 27 scientific staff members at the RIVM, who pilot-tested the revised FIAT-Health. Two publicly reported figures were assessed using the FIAT-Health by three groups of four or five people.

Findings and experiences with assessing the figure were discussed in a plenary session. RG made notes during the discussion, and collected the notes made during the test figure by the participants. The FIAT-Health was adapted according to the feedback received. Consensus on the final version was obtained during a meeting with the sounding board involved in the development of the FIAT-Health. The English version of the FIAT-Health 1.0 was aligned with the changes made to the Dutch version by RG. The revised English version was checked and refined by a native speaker.

Including the potential users, experts, and staff members at the RIVM, a total of 63 participants contributed to the study.

The process of data collection is illustrated in Fig. 1.

## Results

In total 44 potential users were invited and informed on the objective and methods of the study through e-mail. One policy maker, one researcher, three communication officers, and four students declined participation. Three students did not respond. In total 32 people potential users participated in the study. Participants included eight policy makers, eight researchers, eight students, and eight communication officers. All policy makers, researchers, and communication officers had more than 5 years of work experience in their occupation, with the exception of one policy maker and one communication officer who both had less than 3 years of work experience. The potential users worked at the Ministry of Health, Welfare and Sports; the Dutch Healthcare Authority; municipalities; research institutes and universities in the Netherlands. Participating students were graduate students in medicine and public health of whom four were interns at the Amsterdam UMC, location AMC who had no professional relationship with the project team.

### Comparison of potential user and expert assessments

The justifications provided by experts for their assessment resembled all items included in the FIAT-Health, aside from the justification ‘knowledge of the type of methodology’. Potential users using the FIAT-Health 1.0 mentioned as a justification the trustworthiness of the figure, the possibility to verify the content of the figure, and the mentioning of new information in the publicly reported message. These justifications were not mentioned by the experts. Experts used the additional justification of knowledge of type of methodology, and their disapproval of that particular method. One participant also mentioned familiarity with that same method and rated the correctness of the figure negatively, while the participant rated the figure positively. All justifications provided by experts and potential users are listed in Table 1.

**Table 1:** Justifications provided for the final assessment rating by experts and potential users

Justifications provided by both experts and potential users	
• The correctness of the methods	
• Assumptions on which the model is based	
• Match between the primary publication and the reported figure	
• Transparency on the definition of the subject	
• The conclusion that was made based on the results	
• Previous knowledge of the subject	
• Application of the figure in practice	
• An extrapolation was made	
• The geographical area the figure applies to	
• It concerned an estimation	
• No better figures are known about the subject	
• Source of the figure	
• Time period to which the figure relates	
• Match between the population of the reported figure and the primary publication	
• Generalization of the figure	
• Interpretation of the journalist	
• Difference in jargon between the primary publication and the reported figure	
Justifications provided by potential users	
• Credibility of the author	
• Verifiability of the figure	
• New information in the [publicly reported message]	
Justifications provided by experts	
• Method of modelling (the figure has no meaning as the expert considered the construct to be invalid)	

A comparison between the answers by potential users and the experts to the final questions on the correctness of the figure (nr. 14) and the appropriateness of the report (nr. 15) is provided in Fig. 2. Answers were provided on a scale from 1 (negative) to 5 (positive). Participants frequently rated both the correctness of a figure and the appropriateness of the report positively, rating 4 or 5. Experts only provided average [3] or negative (1 or 2) ratings. Potential users rated the correctness of the figures higher or equal to the appropriateness of the report. Experts however, gave the same rating to the correctness of the figure and the appropriateness of its report. Only for figure 4, the overall rating by potential users was lower than the expert rating.

### Evaluation of the FIAT-health 1.0

The topics mentioned by the potential users in the evaluation of the FIAT-Health 1.0 are provided in Table 2. Most frequently, participants from all user groups found the FIAT-Health contributed to their awareness of the main characteristics of the figure due to its structured approach (*n* = 14). This was particularly frequently mentioned by policy makers (*n* = 5). *Policy maker: “In itself it is useful to systematically assess a figure. It does take a lot of time to assess a figure. It forces one to look at the primary publication again.”*

**Table 2:** Topics in the evaluation of the FIAT-Health 1.0, number of times mentioned

Topic	Policy Makers	Researchers	Communication officers	Students	Total
High time investment	4	2	2	1	9
The questionnaire/Excel sheet is complex	5	2	3	1	11
Time investment of checking the primary publication	1	2			3
Reference to the primary publication is helpful	2	1	1		4
The structured way of assessing is good for creating awareness of the characteristics of the figure	5	3	3	3	14
Language is complex	1	3	1	2	7
Questions could be more in-depth	1				1
Goal of the questionnaire is unclear		2		1	3
Explanations clarify the question		1		2	3
Not all questions are relevant for the figure		1	1		2
Unclear meaning primary publication				1	1
The FIAT-Health 1.0 is user friendly	1	1		2	4

Furthermore, the complexity of the FIAT-Health 1.0 was frequently commented on by policy makers, communication officers and researchers (*n* = 11). *Researcher: “I think it is an interesting tool, because it makes you stop and think about the questions you should ask yourself when reading such a report. But I don’t think it is very user friendly, as an Excel file.”* The Excel format of the FIAT-Health 1.0 was evaluated as “*time-consuming” (n = 9)*. Although two students, a policy maker and a researcher thought the FIAT-Health 1.0 was user-friendly (*n* = 4). The language use was considered complicated (*n* = 7), and some potential users (two researchers and one student) could not grasp the goal of the FIAT-Health (*n* = 3). Another topic mentioned in the evaluation was the time investment of checking the primary publications (*n* = 3), while others considered the reference to the primary publication as positive (*n* = 4). Some potential users thought the explanations to the questions (in the Dutch version of the FIAT-Health 1.0) were helpful (*n* = 3).

Potential users recommended the transformation to an online checklist. Furthermore, some potential users commented that not all questions were relevant for the figure they assessed (*n* = 2), or that more in-depth questions regarding for example the methods could be added (*n* = 1). For one participant it was unclear what we meant by ‘primary publication’.

### Consistency of the answers

Out of twenty-six dichotomous questions, seventeen questions were answered consistently among potential users. Nine questions we answered inconsistently.

For the following nine questions two or more potential users answered inconsistently with the majority of answers:

- *3a, Is the figure expressed in absolute terms?*
- *3c, Does the figure you are assessing match the figure in the primary publication?*
- *4b, Does the definition of the subject of the figure you are assessing match the definition of the subject in the primary publication?*
- *5b, Does the definition of the population of the figure you are assessing match the definition in the primary publication?*
- *7a Is the time period in which the units are counted described in the primary publication?*
- *7b, Does the time period to which the figure applies match the time period in the primary publication?*
- *8a, Are the data on which the figure is based collected periodically?*
- *10a, Were the data collected through an existing registration?* and
- 13a, *Was the figure constructed through modelling?*

Analysis of the numerical assessment questions showed a pattern of inconsistency in how potential users responded. On these questions, more than three potential users deviated from the majority. Agreement between potential users’ answers per question per figure for the dichotomous questions is presented in the Appendix: Table 5.

### FIAT-health 2.0

Based on the results of the evaluation the FIAT-Health 1.0 was adapted. The questions that were answered inconsistently or unclear by the potential users were reformulated and the explanations to specific concepts were specified. Most questions that were answered inconsistently were changed into an open-ended question format, while a few questions on the agreement between the primary publication and the reported figure were revised. In addition, the explanation of one question (nr. 13) was extended.

The construct of the FIAT-Health 1.0, namely the overall quantitative assessment of the figure, was replaced by an open-ended answer format. The new construct of the FIAT-Health is aimed at the systematic answering of questions that are important for the interpretation of a figure on health and healthcare and is no longer aimed at constructing an objective quantitative assessment.

Draft versions of the new FIAT-Health 2.0 were tested by scientific staff (*N* = 27) at the RIVM and reviewed by the sounding board. Based on their feedback, final adaptions to the language were made, and the last question [15] was changed to assess the ‘interpretation of the figure’ in the FIAT-Health 2.0, rather than the ‘appropriateness of the report of the figure’ in the FIAT-Health 1.0. The FIAT-Health 2.0 is presented in Table 3. To improve the usability of the instrument a website www.fiathealth.info [28] was created (in Dutch only). On this website, the instrument can be used with a user-friendly interface, with additional functionalities such as the automatic creation of a summary overview of the main characteristics of a figure based on the responses to the questions.

**Table 3:** FIAT-Health 2.0

	FIAT-Health 2.0
What figure would you like to assess? (Provide the phrase in which the figure is mentioned.)	
Question 1. Origin of the figure	
1a.	Is the publication in which the figure is reported a primary publication?^a^
1b.	Is the primary publication known?
1c.	Is the primary publication verifiable?
	*If the primary publication is not accessible, the FIAT-Health cannot be used. Access to the primary publication is necessary to assess the reported figure with the FIAT-Health 2.0.*
1d.	Does the figure you are assessing match the figure in the primary publication?
Question 2. Credibility of the figure	
2a.	Under the responsibility of what institute has the primary publications been published?
2b.	How credible do you consider the author of the primary publication [in relation to this particular figure]?
Question 3. Expression of the figure	
3.	How is the reported figure expressed? *(For example: in absolute terms, percentage, average, fraction)*
Question 4. Subject to which the figure applies	
4a.	To what subject does the reported figure relate?
4b.	Is the subject of the reported figure identical to the subject described in the primary publication?
Question 5. Population to which the figure applies	
5a.	To what population does the reported figure relate?
5b.	Is the population of the reported figure identical to the population described in the primary publication?
Question 6. Geographical area to which the figure applies	
6a.	To what geographical area does the reported figure relate?
6b.	Is the geographical area of the reported figure identical to the geographical area described in the primary publication?
Question 7. Time period to which the figure applies	
7a.	To what time period does the reported figure relate?
7b.	Is the time period of the reported figure identical to the time period described in the primary publication?
Question 8 to 13: Methods of counting and measuring	
	*Multiple methods of counting and measuring are possible.*
Question 8. Data collection	
8.	Are the data on which the figure is based collected only once, periodically or continuously?
Question 9. Sample	
9a.	Is the figure based on a sample?
9b.	What is the sample size?
9c.	What is the response rate?
9d.	Were important groups disregarded in the calculation of the figure? If yes, which groups?
9e.	How do you assess the representativeness of the sample?
Question 10. Registration	
10a.	Were the data collected through an existing registration?
10b.	What registration was used?
10c.	How do you assess the usability of this registration for the calculation of this specific figure?
Question 11. Survey research	
11a.	Were the data collected through survey research?
11b.	Are the questions on which the figure is based described precisely?
11c.	Are the answer categories of the questions described?
11d.	How do you assess the conclusion which was made based on the questions and the answer categories?
Question 12. Direct observations	
12a.	Are the data collected through direct observations?
12b.	How the direct observations take place?
12c.	How do you assess the accuracy of the direct observations?
Question 13. Modelling	
13a.	Was the figure constructed through modelling?
13b.	Are the assumptions which were made in the model known?
13c.	How do you assess the plausibility of the assumptions made in the model?
Final assessment	
How do you assess the correctness of the figure in the primary publication?	
How do you assess the interpretation of reported figure?	

^a^If the figure is assessed in a primary publication, questions that compare the reported figure to the primary publication are passed over i.e. 1d, 4b, 5b, 6b and 7b

The FIAT-Health 2.0 consists of factual questions, questions regarding the agreement between the primary publication and the public report, and open-ended assessment questions. The final assessment of the FIAT-Health 2.0 concerns a description of the correctness of the figure and the interpretation of the public report.

## Discussion

The aim of this study was to test and evaluate the FIAT-Health 1.0 amongst its intended user groups, and further refine the tool based on our results.

Qualitative results indicate that the FIAT-Health supports its users to make similar considerations to experts when they assess a publicly reported figure. The potential users of this study underlined the value of the structured approach of the FIAT-Health in assessing a figure and noted that it made them consider the figure more critically. Furthermore, the FIAT-Health is considered time-intensive and complex by the potential users of this study. The results of this study indicate that it is feasible for potential users to answer factual questions about a figure consistently. Nevertheless, the answers on the quantitative assessment questions were inconsistent.

In line with these results, inconsistently answered and unclear questions of the FIAT-Health 1.0 were rephrased while the consistently answered questions were retained. Most importantly, we revised the underlying construct, in which we assumed that the FIAT-Health can support users in making a quantitative assessment of a figure.

### Limitations

The FIAT-Health 1.0 was tested by its intended users. Because of the time-investment potential users could only assess one figure. As our sample size was small and users did not repeat any measurements, estimates of reliability such as Kappa’s [29] or ideally, Krippendorff’s Alpha [30] could not be calculated.

As we developed the FIAT-Health 1.0, we might have interpreted the results of its evaluation more positively. By reporting our findings, involving potential users outside the researching institute, our preparedness to thoroughly adapt the instrument, and discussing our results with a sounding board outside the project group, we tried to avoid this bias. Furthermore, a risk of selection bias exists due to our purposeful sampling strategy. Those with no interest in using the tool might not have been interested in participating in this study. Seven students declined participation of this study which could indicate that the students might have limited interest in using this tool unless they have a curiosity in healthcare research. Unlike students, policy advisors, communication officers and researchers showed a greater willingness to participate. Consequently, their interest in using a tool to support reporting of figures may be higher.

The evaluation questions were aimed at improving the FIAT-Health, thus potential users focussed on what they thought was unclear and could be amended. The positive sides of the FIAT-Health 1.0 might have been underrepresented in their answers.

One coder has performed the analyses. This might have led to a bias in the coding process, possibly resulting in missed opportunities for the refinement of the tool.

### Context

Most reporting tools and checklists demonstrate a low measure of reliability. Mokkink et al. (2010) found a low inter-rater reliability of the quantitative assessment of the COSMIN Checklist (COnsensus-based Standards for the selection of health status Measurement Instruments) [31]. In addition, Pieper (2017) who performed a review of systematic reviews using the AMSTAR statement (Assessing the Methodological Quality of Systematic Reviews) showed low inter-rater reliability as well [32]. They concluded that an assessment of instruments using only two reviewers would be insufficient in determining reliability, as raters would use their own subjective judgement. Furthermore, dichotomous items are more likely to be answered reliably than scaled questions [33]. It seems to be difficult to construct an objective quantitative assessment of a publication whether it is in science or public communication. Therefore, we consider that in the assessment made using the FIAT-Health, there will always be a certain degree of subjectivity.

While the ratings seemed to be inconsistent, the justifications for assessments of the potential users were closely aligned with the justifications provided by the experts. These results support that the FIAT-Health 1.0 did grasp the right items that support the interpretation of a figure. As policy makers and other users indicated that a structured assessment helped them become more aware of the characteristics of the figure, the primary goal of the FIAT-Health, namely supporting interpretation, was reinforced. When we revised the tool, we aimed to further emphasize this goal. To support users in the assessment of figures on health and healthcare, FIAT-Health 2.0 was revised into a qualitative online appraisal tool consisting of open-ended questions aimed at a better interpretation of publicly reported figures. Both the FIAT-Health 1.0 scoring instrument and 2.0 appraisal tool consists of three types of questions and a final assessment. Questions in the FIAT-Health 1.0 have a closed-ended format, including numerical ratings, while the questions in the FIAT-Health 2.0 primarily have an open-ended format, providing room for descriptive answers and assessments. Both the FIAT-Health 1.0 and 2.0 can be used as a checklist. However, use of the FIAT-Health 2.0 as a checklist is made easier due to its simplified format. The differences between the FIAT-Health 1.0 and 2.0 are described in Table 4.

**Table 4:** Differences between the FIAT-Health 1.0 and 2.0

	FIAT-Health 1.0	FIAT-Health 2.0
Factual questions	Closed-ended questions on the characterization of the figure answered with ‘yes’ or ‘no’.	Includes both open-ended questions on the characterization of the figure, answered by taking information from the public report, and closed-ended questions, answered with ‘yes’ or ‘no’.
Assessment questions	The user gives a rating on a scale from 1 (negative) to 4 (positive) on methodological aspects.	The user describes his or her assessment on methodological aspects providing a numerical rating.
Questions on the primary publication	Questions on the consistency between the publicly reported figure and the figure as described in the primary publication.	Questions on the consistency between the publicly reported figure and the figure as described in the primary publication are rephrased.
Final assessment	The user rates the ‘correctness of the original figure in the primary publication ‘and the ‘appropriateness of the figure in the report’ on a scale from 1 (negative) to 5 (positive).	The user describes his or her considerations on their assessment of the ‘correctness of the figure in the primary publication’, and the ‘interpretation of the reported figure’.
Checklist	Can be used as a checklist.	Use of the FIAT-Health 2.0 as a checklist is made easier due to its simplified format.

Although there are many available checklists and methods to support reporting and assessment of the quality of peer-reviewed scientific publications [14], these checklists that assess statistics in societal publications have not been not tested and constructed scientifically. Studies on the use of checklists in peer-reviewed scientific publications indicate that such a checklist does improve the quality of reporting [34]. For a long time, lay checklists have been published in the form of popular literature, such as Darrel Huffs book “How to Lie with Statistics” [35]. The content of the FIAT-Health 2.0 was constructed systematically. Moreover, the FIAT-Health 2.0 was developed, improved and tested through the involvement of its potential users.

The FIAT-Health 2.0 can contribute to public understanding of statistics in two ways. One, the tool may be used by any person to assess a figure reported in the media. A limitation of this function lies in the construction of the FIAT-Health. We did not have the opportunity to involve the general public in the construction and improvement of the tool, and considering the feedback on the FIAT-Health 1.0, its language might still be difficult to grasp by some. Nevertheless, the tool is publicly available in Dutch and easily accessible online, to be used by those who are interested. Two, the tool is considered useful by policy makers, communication experts and researchers. These are the people that bring statistics under the attention of the public. If they apply the tool to improve their reporting, we may intervene in the communication flows from those creating the figure (research institutes/scientific research) to the receivers (the public) [13]. The figures may be reported more responsibly including a necessary description of sources, construction and methodology. Improved reporting on the most relevant background characteristics of a figure will give the public the information necessary to interpret the reported figure.

### Implications

The potential users of the FIAT-Health have mentioned the usefulness of the tool, indicating that the FIAT-Health would be valuable to the work of policy makers, researchers, and communication officers. Currently, publicly reported statistics are not assessed systematically, but reviewed based on the user’s knowledge and expertise. The FIAT-Health 2.0 can help those without expert knowledge to assess statistics systematically or help researchers and communication officers report findings responsibly. Carefully interpreting statistics is time consuming, thus we recommend development of implementation strategies for those who regularly publish statistics. In its current form, the FIAT-Health 2.0 can be used to create a structured overview of the most important characteristics of a figure, or, when short in time, as a simple checklist. Since using a checklist repeatedly is likely to result in better assessments [33], we recommend people to use the FIAT-Health 2.0 frequently.

## Conclusion

The elements of the FIAT-Health 1.0 were considered useful by the participating policy makers, communication officers and researchers. Expert assessments were comparable to the elements of the FIAT-Health. However, potential users reported the form and language of the tool needed improvement. The tool was refined according to the results of the test and evaluation, transforming the FIAT-Health from a quantitative scoring instrument into an online qualitative appraisal tool. The FIAT-Health 2.0 is a unique instrument that has the potential to help policy makers, communication officers and researchers to systematically assess figures, form a structured interpretation of figures, and aid the better reporting of figures on health and healthcare towards the public.

### Additional file

Additional file 1: FIAT-Health 1.0 in Excel format. (XLSX 64 kb)

## Data Availability

The datasets used and/or analysed in this study are available from the corresponding author on request.

## Appendix

**Table 5:** Agreement per question per figure expressed as number of same answers as part of the total number of given answers

Nr.	Question	Figure 1	Figure 2	Figure 3	Figure 4
1a.	Is the publication in which the figure is reported a primary publication?	7/8	8/8	8/8	8/8
1b.	Is the primary publication known?	7/7	7/8	7/8	5/7^a^
1c.	Is the primary publication verifiable?	7/7	8/8	7/8	4/7^a^
3a.	Is the figure expressed in absolute terms?	4/8^a^	6/8^a^	5/8^a^	6/8^a^
3b.	Is the figure expressed in relative terms?	6/8^a^	6/6	5/5	5/6
3c.	Does the figure you are assessing match the figure in the primary publication?	5/7^a^	6/8^a^	5/8^a^	8/8
4b.	Does the definition of the subject of the figure you are assessing match the definition of the subject in the primary publication?	5/7^a^	6/8^a^	5/8^a^	5/8^a^
5b.	Does the definition of the population of the figure you are assessing match the definition in the primary publication?	6/7	5/8^a^	6/8^a^	5/8^a^
6b.	Does the geographical area of the figure you are assessing match the geographical area in the primary publication?	6/7	5/8^a^	8/8	8/8
7a	Is the time period in which the units are counted described in the primary publication?	8/8	6/8^a^	7/8	5/8^a^
7b.	Does the time period to which the figure applies match the time period in the primary publication?	6/7	4/8^a^	7/8	6/8^a^
8a.	Are the data on which the figure is based collected periodically?	8/8	5/8^a^	6/8^a^	7/8
8b.	Are the data on which the figure is based collected only once?	N.A.	5/5	2/2	1/1
9a.	Is the figure based on a sample?	8/8	5/8^a^	7/8	7/8
9b.	Is the sample size known?	7/8	3/3	1/1	1/1
9c.	Is the response known?	8/8	2/3	1/1	1/1
9d.	Were important groups disregarded in the calculation of the figure?	7/8	2/3	1/1	N.A.
10a.	Were the data collected through an existing registration?	7/8	7/8	5/8^a^	5/8^a^
10b.	Is it known which registration was used?	1/1	7/7	2/3	3/3
11a.	Were the data collected through survey research?	8/8	6/8^a^	7/8	7/8
11b.	Are the questions on which the figure is based described precisely?	7/8	5/6	1/1	1/1
11c.	Are the answer categories of the questions described?	7/8	6/6	1/1	1/1
12a.	Are the data collected through direct observations?	8/8	8/8	8/8	6/8^a^
12b.	Is it known how the direct observations took place?	N.A.	N.A.	N.A.	2/2
13a.	Was the figure constructed through modelling?	7/8	5/8^a^	8/8	6/8^a^
13b.	Are the assumptions which were made in the model known?	1/1	2/3	8/8	3/6^a^

^a^answers are inconsistent (if only one participant answered the question, the consistency rule does not apply)

N.A. not applicable as no answers were given

## Abbreviations

AMC: Academic Medical Center
AMSTAR: Assessing the Methodological Quality of Systematic Reviews
Amsterdam UMC: Amsterdam University Medical Centers
COSMIN Checklist: COnsensus-based Standards for the selection of health status Measurement Instruments
EQUATOR: Enhancing the QUAlity and Transparency Of health Research
FIAT-Health: Figure Interpretation Assessment Tool–Health
GATHER: Guidelines for Accurate and Transparent Health Estimates Reporting
RIVM: The National Institute for Public Health and the Environment (Netherlands)
